# The Effects of Resistant Starch Consumption in Adult Patients With Chronic Kidney Disease: A Systematic Review

**DOI:** 10.1177/20543581221100023

**Published:** 2022-07-12

**Authors:** Kulwant Kingra, Sarah Curtis, Rebecca C. Mollard, Maryam Shamloo, Nicole Askin, Navdeep Tangri, Dylan MacKay

**Affiliations:** 1 Max Rady College of Medicine, Rady Faculty of Health Sciences, University of Manitoba, Winnipeg, Canada; 2 Chronic Disease Innovation Centre at Seven Oaks Hospital, Winnipeg, MB, Canada; 3 Department of Food and Human Nutritional Sciences, Faculty of Agriculture and Food Sciences, University of Manitoba, Winnipeg, Canada; 4 Rady Faculty of Health Sciences, University of Manitoba, Winnipeg, Canada; 5 University of Manitoba Libraries, University of Manitoba, Winnipeg, Canada; 6 Department of Internal Medicine, Section of Nephrology, Seven Oaks Hospital, University of Manitoba, Winnipeg, Canada; 7 Department of Internal Medicine, Section of Endocrinology, Rady Faculty of Health Sciences, University of Manitoba, Winnipeg, Canada

**Keywords:** chronic kidney disease, resistant starch, uremic toxins, oxidative stress, inflammation

## Abstract

**Background:**

Resistant starches (RSs) are not digested by human digestive enzymes and pass through the upper digestive tract to become substrates for colonic bacteria. Resistant starch supplementation has shown promising results in altering the microbiota of animal models of chronic kidney disease (CKD). Resistant starch consumption may influence the production of uremic toxins in CKD.

**Objective:**

To conduct a systematic review to determine whether the consumption of RS reduces the progression of kidney disease in adult patients with CKD.

**Design:**

We included randomized controlled trials comparing RS supplementation to placebo, no treatment, or standard care. Cochrane Central, Embase, MEDLINE, Web of Science, and CINAHL databases were searched. There was no limitation on publication date, but only English manuscripts were included. The search was conducted in July 2020.

**Patients:**

Adult outpatient populations with CKD, using any recognized diagnostic criteria.

**Measurements:**

The primary outcome was change in glomerular filtration rate (GFR) from baseline through the end of the trial in patients not on dialysis; secondary outcomes included change in uremic toxin concentrations (p-cresol/p-cresyl sulfate [p-CS], indoxyl sulfate [IS]) and inflammatory markers (tumor necrosis factor alpha [TNF-α], C-reactive protein [CRP], interleukin 6 [IL-6]) from baseline through the end of the trial, and changes in self-reported symptom scores.

**Methods:**

The Cochrane Collaboration Risk of Bias tool was used to assess risk of bias in included studies. The systematic review results are reported following the Preferred Reporting Items for Systematic Reviews and Meta-Analysis guidelines.

**Results:**

We identified 4 unique studies, reported in 9 publications that met our inclusion criteria, including a total of 215 enrolled participants. Results were calculated using data from the longest reported follow-up time. The primary outcome of changes in kidney function markers was only studied in 1 trial; this trial reported an increase in creatinine and a decrease in blood urea nitrogen; no changes in GFR were reported. A review of the secondary outcomes showed an overall decline in IS, TNF-α, and IL-6, in RS groups, but with mixed results in p-CS and CRP/high-sensitivity CRP. Safety data showed that RS was well tolerated with no reports of excessive side effects.

**Limitations:**

We determined a meta-analysis was not feasible due to clinical heterogeneity between study populations and differences in reported outcomes in the included studies.

**Conclusion:**

There is limited and inconsistent evidence on the impact of RS in adult patients with CKD. Further research is needed to determine the safety and efficacy of RS supplementation in this population.

## Abrégé

**Contexte:**

Les amidons résistants ne sont pas dégradés par les enzymes digestives humaines; ils traversent le tube digestif supérieur et deviennent des substrats pour les bactéries du côlon. La supplémentation en amidons résistants a montré des résultats prometteurs dans la modification du microbiote chez les modèles animaux d’insuffisance rénale chronique (IRC). Dans ce contexte, la consommation d’amidons résistants pourrait avoir une incidence sur la production de toxines urémiques.

**Objectif:**

Procéder à une revue systématique afin de déterminer si la consommation d’amidons résistants limite la progression de la maladie chez les adultes atteints d’IRC.

**Conception de l’étude:**

Ont été inclus les essais contrôlés randomisés qui comparaient la supplémentation en amidons résistants à un placebo, à l’absence de traitement ou aux soins courants. La recherche a été effectuée en juillet 2020 dans les bases de données Cochrane Central, Embase, Medline, Web of Science et CINAHL. La date de publication n’était pas limitée, mais seuls les articles rédigés en anglais ont été inclus.

**Sujets:**

Des adultes atteints d’IRC et suivis en ambulatoire, selon tout critère de diagnostic reconnu.

**Mesures:**

Le principal critère d’évaluation était un changement dans le taux de filtration glomérulaire entre le début et la fin de l’essai chez les patients non dialysés. Les résultats secondaires comprenaient un changement, entre le début et la fin de l’essai, dans les concentrations de toxines urémiques (sulfate de p-crésol/crésyle, sulfate d’indoxyle) et de marqueurs d’inflammation (TNFα, CRP, IL-6), ainsi que dans les scores de symptômes auto-déclarés.

**Méthodologie:**

L’outil de Risque de Biais de la Collaboration Cochrane a été utilisé pour évaluer le risque de biais dans les études incluses. Les résultats de la revue systématique sont présentés conformément aux directives PRISMA.

**Résultats:**

Quatre études uniques, présentées dans neuf publications et portant sur un total de 215 patients, répondaient à nos critères d’inclusion. Les résultats ont été calculés à partir des données de la plus longue période de suivi rapportée. Un seul de ces essais avait examiné le critère principal d’évaluation, soit un changement dans les marqueurs de la fonction rénale. On y rapportait une augmentation du taux de créatinine et une diminution du taux de l’urée dans le sang. Aucun changement dans le débit de filtration glomérulaire n’avait été signalé. Un examen des résultats secondaires a révélé une diminution globale du sulfate d’indoxyle, du TNF alpha et d’IL-6 dans les groupes de patients qui prenaient des suppléments d’amidon résistants, mais des résultats mitigés en ce qui concerne les taux de p-CS et de CRP/hsCRP. Les données de sécurité montraient que les amidons résistants étaient bien tolérés, aucun effet secondaire important n’ayant été rapporté.

**Limites:**

Une méta-analyse n’était pas réalisable en raison de l’hétérogénéité clinique entre les populations étudiées et des différences dans les résultats rapportés dans les études incluses.

**Conclusion:**

Les données sur les effets d’une supplémentation en amidons résistants chez les adultes atteints d’IRC sont limitées et manquent d’uniformité. D’autres recherches examinant la sécurité et l’efficacité d’une supplémentation en amidons résistants dans cette population sont nécessaires.

## Introduction

Chronic kidney disease (CKD) is a pervasive condition affecting ~13% of the general population and ~36% of high-risk population.^1^ As kidney function declines, electrolytes, excess fluid, and nitrogen-based waste products accumulate in the body contributing to the advancement of the disease and associated complications. Increasing evidence suggests that therapeutic interventions aimed at fortifying the intestinal microbiota may slow the progression of CKD.^2^

The human gastrointestinal microbiota plays a fundamental role in the overall health status of the host. Considering a symbiotic “supplementary organ,” the composition and metabolic activity of these microbial communities are dependent on host genome, diet, health, and lifestyle factors. Whereas healthy individuals benefit from the protective and trophic functions associated with a balanced microbial ecosystem, dysbiosis (an altered microbiome) has been shown to contribute to various pathophysiologies. In patients with CKD, dysbiosis is associated with inflammation, oxidative stress, and a higher production of reactive oxygen species (ROS). Together, these factors induce kidney damage by impacting microcirculation and blood perfusion leading to further renal tubular injury.^2,3^

Resistant starches (RSs), such a green banana, raw potato, or high-amylose maize starch, contain linkages which are resistant to hydrolysis in the human small intestine.^2^ Resistant starches pass through the upper digestive tract to the colon, where they act as a digestible substrate for beneficial colonic bacteria.^2^ Supplementation with RS has shown promising results in altering the microbiota of animal models of CKD. Human studies involving RS supplementation suggest that it can alter the human gut microbiota^2,3;^ however, this has not yet been shown in people with CKD.^2^ The products of RS fermentation in the colon include gases (methane, hydrogen, carbon dioxide) and important metabolites such as short-chain fatty acids (SCFAs). It is hypothesized RS could potentially reduce the amount of nitrogenous uremic toxins known to accumulate in patients with CKD.^2^ Generally low cost and readily available, RS may become an important component of the treatment regimen for patients with CKD. Thus, the main objective of this systematic review is to assess the efficacy and tolerability of RS in slowing the progression or delaying symptoms in adult patients with CKD.

## Methods

This systematic review was conducted using the Methodological Expectations of Cochrane Intervention Reviews guidelines. We followed the Preferred Reporting Items for Systematic Reviews and Meta-Analysis (PRISMA) guidelines to report our results.^4^ A completed PRISMA checklist is provided in the Supplemental Material. Our objective was to synthesize evidence addressing this question: Does the consumption of RS slow the progression of kidney disease in adult patients with CKD?

### Search Strategy and Study Selection

A knowledge synthesis librarian developed a search strategy for MEDLINE, which was independently reviewed by a second librarian using the PRESS checklist (Supplemental Appendix Table S1).^5^ This search strategy was modified and adjusted for use in other databases including Cochrane Central, Embase, Web of Science, and CINAHL. These databases were searched since their inception. The search strategy was performed in July 2020 and references were managed using Rayyan (Rayyan, Doha, Qatar).^6^ In late July of 2020, 2 reviewers (S.C. and K.K.) independently screened titles and abstracts to determine if the study met the inclusion criteria or if they were deemed ineligible due to the exclusion criteria (Supplemental Appendix Table S2). We included randomized controlled trials (RCTs) that involved adult outpatients (>18 years of age) with CKD comparing RS supplementation to placebo, no treatment, or standard care. Chronic kidney disease was defined as glomerular filtration rate (GFR) <60 mL/min/1.73 m^2^ or having markers of kidney damage with duration >3 months, including people on dialysis. There were no limitations on publication year, but we required full-text manuscripts in English for feasibility. Each report was classified as follows: “include,” “maybe,” or “exclude.” All reports classified as “include” or “maybe” by either reviewer were retrieved for full text review. Disagreements were resolved by discussion between the 2 reviewers with third-party adjudication when necessary.

Our primary outcome was reduced decline in GFR from baseline through the end of the trial in patients not on dialysis. Our secondary outcomes included reduced uremic toxin buildup (p-cresol/p-cresyl sulfate [p-CS], indoxyl sulfate [IS]) from baseline through the end of the clinical trial; reduced inflammatory markers in kidney disease (tumor necrosis factor alpha [TNF-α], C-reactive protein [CRP], interleukin 6 [IL-6]) from baseline through the end of the clinical trial; and improvement in self-reported symptom scores.

### Data Abstraction and Management

Data points were independently extracted by 2 reviewers (S.C. and K.K.) from all included trials using a standardized form developed in MS Excel 2019 (Microsoft Corporation, Redmond, Washington).^7^ Disagreements were resolved through discussion between the 2 reviewers with third-party adjudication when necessary. The following data points were extracted: author, year of publication, year of study, country, setting, population demography, participant characteristics (age, sex distribution, health, and socioeconomic status), name, type, method, measurement and duration of intervention, control group, change in biomarkers for both intervention and control groups including: estimated glomerular filtration rate (eGFR), creatinine, blood urea nitrogen (BUN), p-CS, IS, TNF-α, CRP, IL-6, self-reported symptom scores, and tolerability of RS. We attempted contacting authors of all included trials for which relevant outcome data were missing or reported but not extractable (eg, data presented as an illustration). In the event of multiple companion reports of an included trial, we used the one that had the most complete data set as the primary report and listed all other publications as secondary reports.

### Risk of Bias Assessment

Two reviewers (S.C. and K.K.) assessed the internal validity of included trials using the Cochrane Collaboration Risk of Bias tool.^8^ The overall risk of bias for each trial was based on the adjudication of 5 individual domains: bias arising from the randomization process, bias due to deviations from the intended interventions, bias due to missing outcome data, bias in measurement of outcome, and bias in the selection of reported results. Each domain was rated “low risk,” “some concerns,” or “high risk” (Supplemental Appendix Table S7). Disagreements were resolved through discussion between the 2 reviewers with third-party adjudication when necessary.

## Results

### Search Results

**Figure 1.**
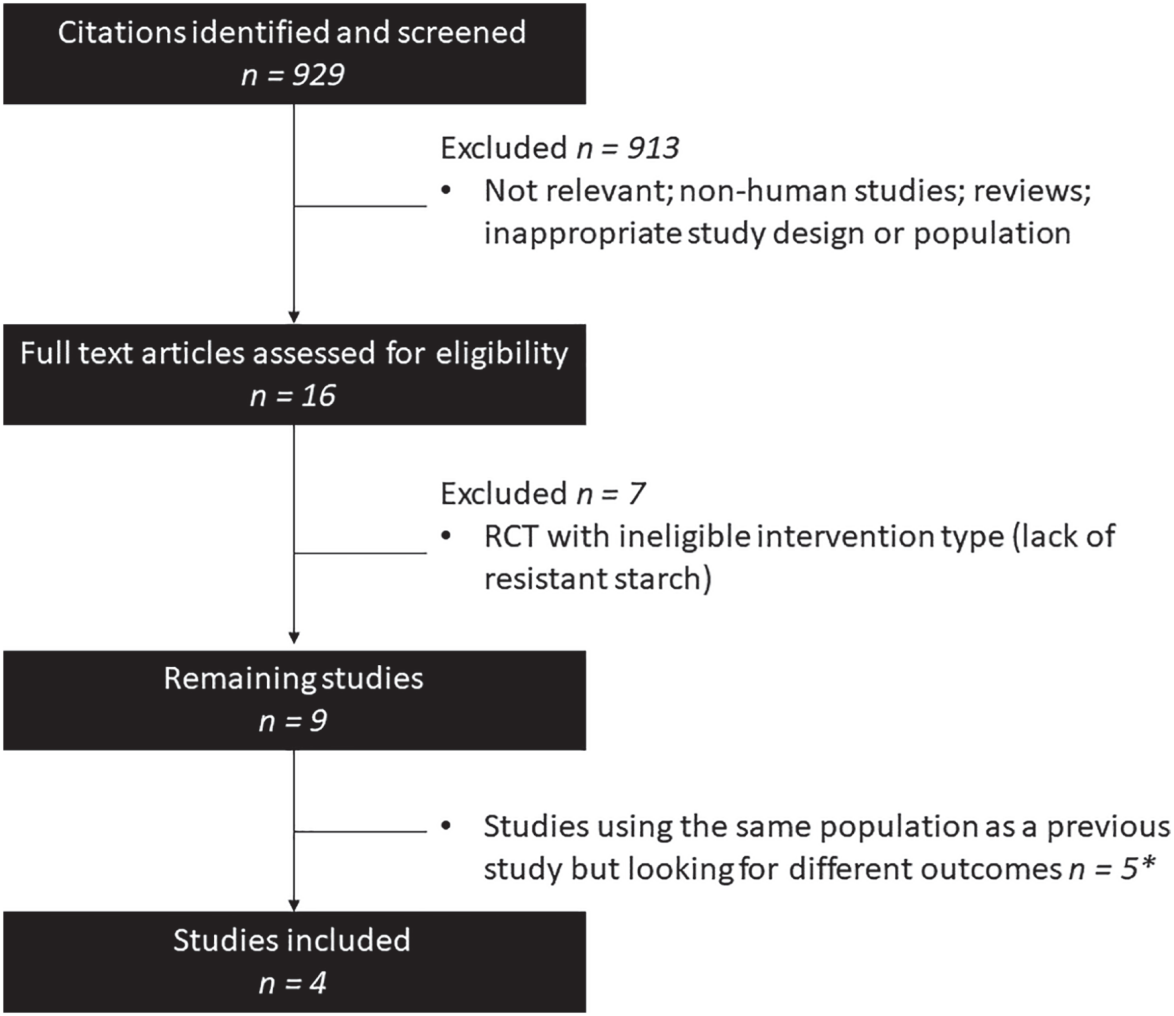
PRISMA flow diagram. *Note.* RCT = randomized controlled trial; PRISMA = Preferred Reporting Items for Systematic Reviews and Meta-Analysis. *Three follow-up studies done in Brazil used the same population but looked at different outcomes and thus were only counted as 1 study in the final tally. The study with the longest reported follow-up time was documented for inclusion. Two follow-up studies done in Iran used the same approach as the Brazilian studies. The original study was documented for inclusion.

From 929 identified citations, 6 non-English studies were identified by the search, and their abstracts were reviewed as well (2 Russian, 2 Portuguese, 1 Spanish, 1 Turkish); none of these abstracts were for RCTs and were excluded. Sixteen potentially eligible full-text articles were reviewed and 9 met our inclusion criteria. Of these, 5 reported different outcomes from the same study population, leaving 4 unique studies to be analyzed. The study report with the longest follow-up time was documented for inclusion (Figure 1).

### Study Characteristics

Characteristics of the included studies are summarized in Table 1. Included trials were conducted in the United States,^9^ Iran,^10^ China,^11^ and Brazil^12^ between 2014 and 2020. Average participant age ranged between 40 and 74 years, with a higher proportion of males in Sirich et al^9^ (24/40), Tayebi Khosroshahi et al^10^ (28/44), and Meng et al^11^ (39/70). Esgalhado et al^12^ did not specify the distribution of male and female participants within the study. Four trials included a total of 215 enrolled participants above the age of 18 years: 140 patients with kidney failure receiving hemodialysis treatment^9,10,12^ and 75 patients with diabetic kidney disease not receiving dialysis.^11^

**Table 1.** Summary of Randomized Controlled Trials That Evaluated Resistant Starch Use in Patients With CKD.

Study,^a^ country	Study size start (end)	Sex (M/F)	Patient population	Age (mean ± SD; years)	Duration (weeks)	Type of RS (specific product)	RS dose	Comparator(s)	Kidney function markers	Uremic toxins	Inflammatory markers	Safety/tolerability
Sirich et al,^9^ United States	56 (40)	T: 11/9 C: 13/7	HD	A: 54 ± 14 B: 58 ± 13	6	Type 2 RS (Hi-Maize 260 powder)	15 g/d × 1 w, 30 g/d × 5 w	Waxy corn starch	N/A	•	•	•
Tayebi Khosroshahi et al,^10^ Iran	46 (44)	T: 12/10 C: 16/6	HD	A: 52 ± 11 B: 60 ± 14	8	Type 2 RS (HAM-RS2 Biscuits)	20 g/d × 4 w, 25 g/d × 4 w	Regular wheat flour biscuits	N/A	•	•	•
Meng et al,^11^ China	75 (70)	T: 18/16 C: 21/15	Nondialysis CKD	A: 62.85 ± 9.3 B: 61 ± 9.5	12	Type 2 RS (High-RS, low-protein flour)	50 g twice daily with lunch and supper × 12 w	Protein-restricted diet with common staple	•		•	
Esgalhado et al,^12^ Brazil	38 (26)	Not specified	HD	A: 56.0 ± 7.5 B: 53.5 ± 11.4	12	Type 2 RS (Hi-Maize 260 powder)	16 g/d × 4 w, washout × 4 w, control × 4 w (reversed in control group, crossover design)	Manioc flour	N/A	•	•	

*Note*. M = male; F = female; RS = resistant starch; HD = hemodialysis; HAM-RS2 = high-amylose maize-resistant starch type 2; Hi-Maize 260 = high-amylose maize starch (Ingredion, Illinois, USA); w = weeks; d = day; T = treatment group; C = control group; N/A = not applicable; • = was measured; CKD = chronic kidney disease.

a Sorted by year of publication.

The trial duration ranged from 6^9^ to 12 weeks.^11,12^ All of the intervention groups used type 2 RS; 2 trials used Hi-Maize 260 powder (high-amylose maize starch [Ingredion]),^9,12^ 1 trial used high-amylose maize RS type 2 biscuits,^10^ and 1 used high-RS low-protein flour.^11^ Comparators were waxy corn starch,^9^ regular wheat flour biscuits,^10^ protein-restricted diet with common staple,^11^ and manioc flour.^12^

### Data Synthesis and Risk of Bias Assessment

The results extracted from studies identified in the search were summarized descriptively. It was not methodologically appropriate to pool results via meta-analysis, due to the limited number of eligible studies and their heterogeneity in terms of population and outcomes. In terms of risk of bias, 2 of the studies were rated overall as “low” and 2 studies were rated overall as “some concerns” (Supplemental Appendix Table S7). Despite being rated “low” by the risk of bias tool, we had concerns about the units reported in the study by Esgalhado et al^12^ and were not able to contact the authors to discuss our concerns, therefore we remain cautious in terms of this study’s results. In addition, none of the studies reported change in eGFR as an outcome, although they likely could have. A review of the 3 trials with registries did not suggest any evidence of outcome reporting bias; however, the study by Meng et al^11^ did not have a registry, so the potential for outcome reporting bias for this trial cannot be ruled out.

### Change in Kidney Function

The study by Meng et al^11^ was the only study involving patients with kidney disease not yet receiving renal replacement therapy. The study did not report any changes to GFR but instead focused on creatinine, BUN, and urinary albumin-to-creatinine ratio (UACR). The study by Meng et al^11^ showed a slight increase in creatinine by +0.0053 ± 0.0105 mg/dL, while BUN and UACR were not different with RS consumption compared with control. The magnitude of this change in creatinine is not clinically significant.^13^

### Change in Uremic Toxins and Inflammatory Markers

We discovered errors in the reports on the units of uremic toxins and inflammatory markers in some of the studies. We were able to confirm this after contacting an author who was involved with the studies from Iran.^10,15,16^ Indoxyl sulfate and p-CS were corrected to mg/L, TNF-α and IL-6 were adjusted to pg/mL, and CRP was changed to mg/L. We attempted to contact the authors of the study by Esgalhado et al^12^ due to a possible error recording the units of IL-6 but we did not receive a response. We adjusted the units of IL-6 to pg/mL because that is how the units were originally recorded in the pilot study,^14^ which showed results for the first half of the crossover trial.

Three of the 4 included trials reported changes in uremic toxins (p-CS, IS; Table 2). While the majority of groups across trials showed a decline in p-CS, the crossover trial by Esgalhado et al^12^ reported an increase of +21.00 ± 20.71 mg/L in patients that received RS first followed by washout and control. All 3 trials showed a reduction in IS with the study by Sirich et al^9^ and Esgalhado et al^12^ both reporting statistically significant results, −5 ± 1 mg/L (total p-CS) and −1.3 ± 3.6 to −10.99 ± 5.00 mg/L, respectively.

**Table 2.** Change in Uremic Toxins From the Randomized Controlled Trials Evaluating Resistant Starch Use in Patients With Chronic Kidney Disease.

Study	Change in p-CS,^a^ mg/L	Change in IS,^a^ mg/L
Sirich et al^9^	−3 ± 1.5 (total p-CS; study also reported free levels)	−5 ± 1 (total IS; study also reported free levels)
Tayebi Khosroshahi et al^10^	−2.5^b^ (Khosroshahi et al^15^)	−3.40^b^ (Khosroshahi et al^15^)
Esgalhado et al^12^	RS first, followed by 4-week washout, then control: +21.00 ± 20.71 Control first, followed by 4-week washout, then RS: −7.2 ± 18.2	RS first, followed by 4-week washout, then control: −10.99 ± 5.00 Control first, followed by 4-week washout, then RS: −1.3 ± 3.6

*Note*. p-CS = p-cresol sulfate/p-cresyl sulfate (reported differently in different trials); IS = indoxyl sulfate; RS = resistant starch.

a Measure described is the change in biomarker intervention group minus control group.

b SD not reported (interquartile range reported in study).

While all trials reported changes in inflammatory markers (TNF-α, CRP, IL-6), results were contradictory (Table 3). The studies by Tayebi Khosroshahi et al^10,15^ showed reductions in TNF-α across groups in contrast to an overall increase (+5.2 ± 0.52 pg/mL) seen in study by Meng et al.^11^ Esgalhado et al^12^ reported a statistically significant decrease (−1.8 mg/L) in high-sensitivity CRP (hsCRP) in the RS group similar to reductions reported by Sirich et al^9^ (−1.0 ± 1.00 mg/L). In contrast, CRP increased in the trial done by Tayebi Khosroshahi et al.^10,15^ IL-6 increased among participants consuming RS compared with control within Meng et al,^11^ but there was a reduction ranging between −5.93 ± 8.68^12^ and −71.34 ± 18.2 pg/mL^15^ within the other trials.

**Table 3.** Change in Inflammatory Markers From the Randomized Controlled Trials Evaluating Resistant Starch Use in Patients With Chronic Kidney Disease.

Study	Tumor necrosis factor alpha,^a^ pg/mL	C-reactive protein,^a^ mg/L	Interleukin 6,^a^ pg/mL
Sirich et al^9^	N/A	−1.0 ± 1.0	N/A
Tayebi Khosroshahi et al^10^	−158.83 ± 5.31 (Tayebi Khosroshahi et al^10^)	+0.35 ± 0.263^c^ (Tayebi Khosroshahi et al^10^)	−71.15 ± 3.427 (Tayebi Khosroshahi et al^10^)
	−125.71 ± 38.06 (Laffin et al^16^)	+4.0^b, c^ (Khosroshahi et al^15^)	−71.34 ± 18.2 (Laffin et al^16^)
Meng et al^11^	+5.2 ± 0.52	N/A	+1.3 ± 0.14
Esgalhado et al^12^	N/A	−1.8^b, c^ (Esgalhado et al^14^)	RS first, followed by 4-week washout, then control: −17.69 ± 3.18 Control first, followed by 4-week washout, then RS: −5.93 ± 8.68

*Note*. N/A = not applicable; RS = resistant starch.

a Measure described is the change in biomarker intervention group minus control group.

b SD not reported (interquartile range reported in study).

c Reported as high-sensitivity C-reactive protein in study.

### Quality of Life and Tolerability

Self-reported symptom scores based on the Kidney Disease and Quality of Life Questionnaire were completed in the study by Sirich et al^9^ and 2 of the studies by Tayebi Khosroshahi et al.^10,15^ The symptom scores reported with RS consumption were not different from controls. Resistant starch was shown to be well tolerated and no excessive side effects were reported (Table 4).

**Table 4.** Summary of Safety and Tolerability Outcome Information From the Randomized Controlled Trials Evaluating Resistant Starch Use in Patients With Chronic Kidney Disease.

Study	Self-reported symptom scores (Kidney Disease and Quality of Life Questionnaire)	Tolerability of resistant starch
Sirich et al^9^	Well tolerated with no significant change in either group −1 ± 1^a^	
Tayebi Khosroshahi et al^10^	Well tolerated with no significant change in either group (No numerical data provided; Tayebi Khosroshahi et al^10^)	No excessive side effects reported (Khosroshahi et al^15^)
	Well tolerated with no significant change in either group (No numerical data provided; Khosroshahi et al^15^)	

a Measure described is the change in biomarker intervention group minus control group.

## Discussion

In this review, we summarized 4 clinical trials exploring the therapeutic efficacy of RS supplementation in adult patients with CKD. We found only one trial studied the effects of RS in kidney function and symptoms in the nondialysis CKD population with no evidence of benefit in this population. When we included patients on hemodialysis, similar discrepancies were found between studies reporting uremic toxin levels (p-CS, IS) and inflammatory markers (TNF-α, CRP, IL-6), although most studies reported overall declines in uremic toxin levels following consumption of RS. Discrepancies between trials reporting inflammatory markers were more pronounced with little consensus between the included reports. However, despite the inconsistencies, the overall magnitude of changes reported in the uremic toxins and inflammatory markers in these 4 trials was generally small. It is also difficult due to the short duration of these trials to determine whether these changes could be clinically important.

Our primary outcome focused on changes in kidney function markers. Unfortunately, there was only one trial which evaluated this outcome in a nondialysis population, and we were therefore unable to make any conclusions about efficacy or safety. Our secondary outcomes included changes in uremic toxins following RS consumption. As stated previously, research suggests one way RS consumption may improve kidney function or symptoms of kidney failure is by lowering the production of uremic toxins in the gut.^17^ Many uremic toxins, such as IS and p-CS, are produced exclusively by the gut microbiome^18^ through the proteolytic digestion of aromatic amino acids (tyrosine and tryptophan, respectively).^19^ Serum concentrations of these toxins are significantly elevated in patients with CKD and are strongly associated with disease progression.^20^ This accumulation is largely due to low dialytic clearance^21^ and increased concentrations of bacterial families possessing indole- and p-cresol–forming enzymes (ie, Clostridiaceae and Enterobacteriaceae).^22^ High concentrations of IS and p-CS have been associated with various clinical symptoms, including uremic pruritus,^23^ and cardiovascular mortality in patients with CKD.^24-28^ Recent attempts to pharmacologically bind uremic toxins in the gut with activated charcoal have been unsuccessful in clinical trials,^27^ perhaps because the binding was not effective, or because the burden on patients (30 pills per day) was too much to maintain compliance. This review showed that RS may reduce uremic toxins such as p-CS and IS but to what degree remains unclear. While all included trials reported significant reductions in IS, values varied. Results were similar for p-CS, except for the unexpected result reported by Esgalhado et al^12^ where p-CS values increased following a washout and control period.

In addition to uremic toxins, we aimed to present results showing the impact of RS on inflammatory markers and any potential association between the two. Reducing the production of uremic toxins and increasing SCFA production through consumption of RS may decrease ROS production which in turn inhibits the body’s inflammatory response. In the current study, Esgalhado et al^14^ reported decreases in hsCRP and IL-6 with the authors noting a positive correlation between differences (Δ) of IL-6 and IS. In addition, the study by Tayebi Khosroshahi et al^10^and Laffin et al^16^ showed reductions in TNF-α across groups. These results are reflected in a study by Rossi et al^29^ that reported an independent association between IL-6 and both free and total IS and p-CS in a cohort of 149 patients with stages 3 and 4 CKD. In addition, the authors reported positive associations between free and total IS and TNF-α. These findings are consistent with previous in vitro studies demonstrating that both IS and p-CS stimulate IL-6 gene expression and IS stimulates TNF-α expression.^30^

A similar review by Jia et al^31^ examined the effects of type 2 RS in patients with kidney failure undergoing hemodialysis and conducted a meta-analysis. However, it is our understanding that they meta-analyzed data from the same heterogeneous study populations and may have included incorrectly reported data, as mentioned in the “Results” section. We contacted one of the authors of the studies who provided us with the raw data and confirmed that the units were incorrectly reported in the published manuscripts. In addition, our literature search strategy differed from Jia et al, by including all types of RS as well as patients not on dialysis.

The strengths of this review include our adherence to the Methodological Expectations of Cochrane Intervention Reviews guidelines and an a priori registered protocol, as well as our peer-reviewed search strategy. In addition, we evaluated study quality using the Cochrane Collaboration Risk of Bias tool. One potential limitation of this review is that our search strategy could have missed studies that were not specifically focused on CKD, but whose population may have contained some participants with CKD. However, the chances of this are unlikely as the search strategy should have identified any studies involving kidney disease, so any study who identified having participants with kidney disease should have been captured. In addition, our search strategy did not include gray literature or conference abstracts, so it may have been possible that existing studies were not captured, especially if they were only presented at conferences and not published in manuscripts.

Our review highlights the paucity of research examining the effects of RS supplementation among patients with CKD, especially those not yet on dialysis. The studies identified in this review contained small sample sizes and were of moderate to low methodological quality. Higher quality and longer term trials are needed to understand the impact of RS supplementation on clinical outcomes in CKD patients. Clinical trial work that compares different types of RS and examines the impact of dose is needed. In addition, trials that look at RS in earlier stages of CKD are needed to investigate their potential on disease progression. These trials should measure an established marker of kidney function, such as eGFR, as well as uremic toxin concentrations and inflammatory markers. Ultimately, larger multicenter RCTs using RS focused on CKD progression in predialysis populations, and quality of life and mortality in dialysis populations, will need to be conducted before RS consumption could be considered a legitimate therapy in CKD.
